# Contrast-enhanced ultrasound diagnosis of prostatic sarcoma: Two case reports

**DOI:** 10.1097/MD.0000000000024038

**Published:** 2021-01-15

**Authors:** Guozhu Wu, Ran Sun, Hua Hong, Yumin Wang, Jing Li, Qian Liu, Aitong Sun

**Affiliations:** Department of Ultrasound Medicine, Inner Mongolia Autonomous Region People's Hospital, Hohhot, Inner Mongolia, 010017, China.

**Keywords:** contrast-enhanced ultrasonography, prostate, prostate sarcoma, prostate-specific antigen

## Abstract

**Rationale:**

Prostatic sarcoma (PS) is a very rare malignant tumor that accounts for <0.1% of prostate malignancies, and Ewing's sarcoma is an extremely rare form of PS.

**Patient concerns:**

We reported on a 64-year-old patient with PS and a 36-year-old patient with Ewing's sarcoma, both of whom were examined by contrast-enhanced ultrasonography (CEUS) before surgery.

**Diagnoses:**

The 2 cases were proven to be prostatic stromal sarcoma, which was confirmed by imaging manifestations and histopathological findings.

**Interventions:**

The 64-year-old patient underwent radical prostatectomy, and the 36-year-old patient underwent chemotherapy combined with local radiotherapy.

**Outcomes:**

PS showed diffuse enlargement of the prostate on sonography, and the necrotic liquefying area within the large vessels could be clearly displayed by CEUS. CEUS can be advocated as a valuable noninvasive and safe imaging diagnosis method for PS.

## 1 Introduction

Prostate cancer is the second most common cancer worldwide, with 1,276,106 new cases in 2017.^[1]^ Prostatic sarcoma (PS) is a rare stromal tumor, accounting for less than 0.1% of primary malignant prostate tumors in adults.^[2]^ PS is generally seen in patients between 25 and 86 years old, half of whom are younger than 50 years old.^[3]^ Ewing's sarcoma is an extremely rare PS that mostly occurs in children and young adults,^[4]^ and few cases have been reported thus far.^[4–7]^ In addition, the clinical manifestations of PS are not specific. Imaging examination by computed tomography (CT) or magnetic resonance imaging (MRI) can provide clues for the detection of PS; however, there are no specific diagnostic criteria.^[8]^ Ultrasound diagnosis of PS has rarely been reported.^[9]^ To the best of our knowledge, contrast-enhanced ultrasound (CEUS) has not been used for the diagnosis of PS in the literature to date. In this case report, we described 2 cases of PS and discussed the significance of CEUS features in the diagnosis of PS.

## 2 Case report 1

### 2.1 Clinical data

A 64-year-old man was referred to our hospital and presented with dysuria for 2 months. Since the onset of the disease, there had been no incidences of low fever or night sweat and no significant changes in weight. Nevertheless, he complained about a decline in physical strength since the onset of the disease. He had a history of hypertension and diabetes for more than 20 years and had a cerebral infarction 8 years ago. On physical examination, a grade 3 enlarged prostate was found by digital rectal examination, with a hard texture, rough surface, and poor mobility. On laboratory examination, all other routine blood indexes were within the normal limits. The prostate-specific antigen (PSA) value was 0.09 ng/mL, which was in the normal range (<4.0 ng/mL), and other tumor markers, including carcinoembryonic antigen and glucosaminidase, were also within the normal limits. Urinalysis showed that all indicators were in the normal range. Based on the details above, we performed transabdominal contrast-enhanced ultrasonography and transrectal prostate biopsy.

### 2.2 Imaging findings

**Figure 1:**
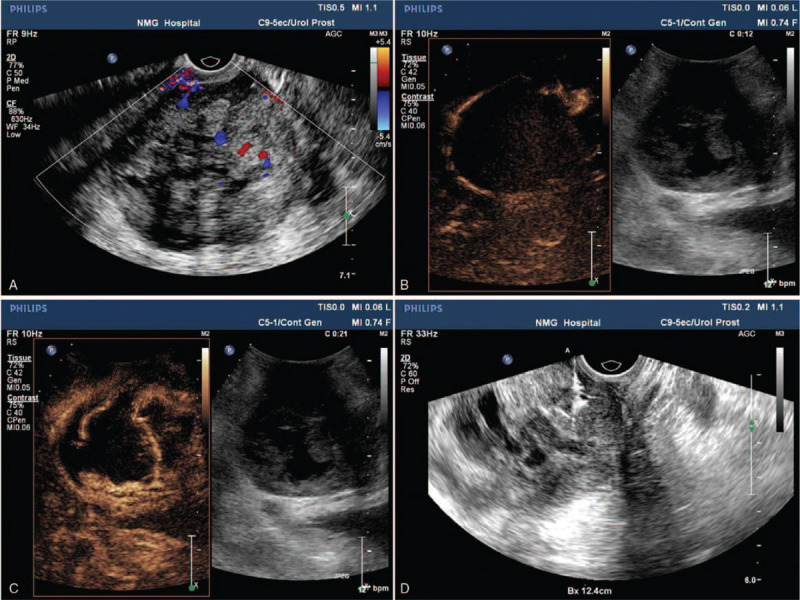
US imaging findings of the PS. Conventional US reveals a multiloculated soft tissue mass in the prostate within rich blood supply signals from the surrounding soft tissue (A). CEUS shows that the peritumoral parenchyma began to enhance rapidly in 12 s (B) and large blood vessels in the necrosis liquefying area of the tumor center (C). According to the enhanced area shown by CEUS, a biopsy was performed (D). CEUS = contrast-enhanced ultrasonography, PS = prostatic sarcoma, US = ultrasonography.

Transrectal ultrasound showed that the appearance of the prostate was enlarged, the size was 91 mm × 63 mm × 90 mm, the outline was irregular, the internal echo was not uniform, and a necrotic liquefying area in the center was seen, with a range of approximately 78 mm × 48 mm; furthermore, color Doppler flow imaging (CDFI) revealed rich blood supply signals from the surrounding soft tissue (Fig. 1A), RI: 0.89. CEUS of the abdomen showed that the peritumoral parenchyma began to enhance rapidly in 12 seconds (Fig. 1B), the large blood vessels in the necrotic liquefying area of the tumor center enhanced synchronously with the surrounding tissues, and the enhancement was most significant at 21 seconds (Fig. 1C). The necrotic area shown by contrast-enhanced ultrasonography was much larger than that by two-dimensional ultrasonography. According to the enhanced area as shown by CEUS, a biopsy was performed (Fig. 1D), and the tissue strip was sent to pathology. The patient was pathologically proven to have prostatic stromal sarcoma.

### 2.3 Operation and treatment

The pathological and immunohistochemical results showed that the patient had stromal sarcoma of the prostate. He underwent radical prostatectomy because of a lack of obvious metastasis, which was supported by PET/CT. After the operation, he had a catheter and abdominal drainage tube installed. One month later, the amount of drainage fluid increased. Urinalysis showed that the white blood cell, red blood cell, epithelial cell, and bacterial counts in the urine had increased. After nutritional support and symptomatic treatment, the patient's condition improved, and chemotherapy combined with local radiotherapy was gradually administered. However, the patient died 1.5 years after surgery.

## 3 Case report 2

### 3.1 Clinical data

**Figure 2:**
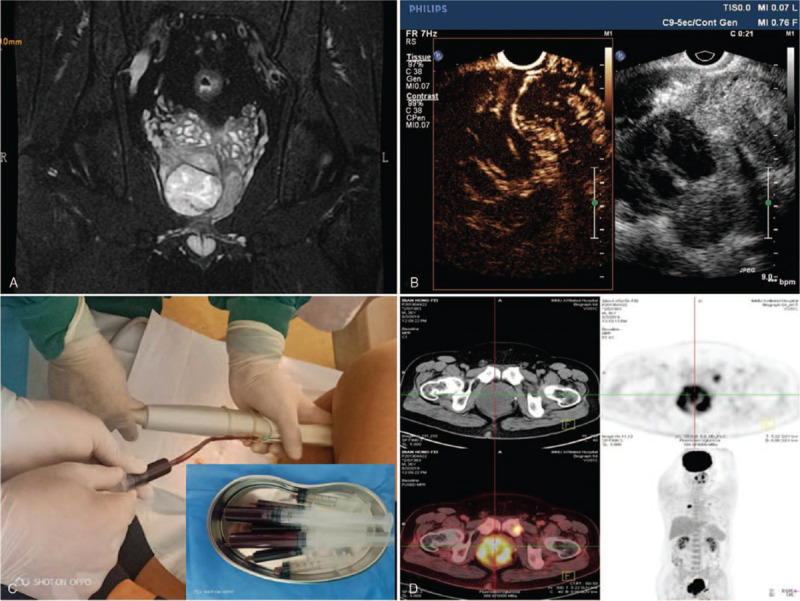
Imaging findings of Ewing's sarcoma. MRI revealed that the prostate was enlarged and the internal signal was uneven. A necrotic cystic area with long T1 and T2 signals was seen in the center (A). TR-CEUS showed that the peritumoral parenchyma enhanced rapidly and that the large blood vessels in the necrotic liquefying area enhanced rapidly and synchronously with the prostate parenchyma (B). According to the enhanced area shown by CEUS, a biopsy was performed, and 65 mL of dark red plasma was drawn out (C). PET/CT showed that the patient had bone metastases in the left iliac bone (D). MRI = magnetic resonance imaging, TR-CEUS = transrectal contrast-enhanced ultrasonography.

A 36-year-old male presented with frequent and weak urination for 2 months. Therefore, the patient underwent an MRI scan in a local hospital, which showed that the prostate was enlarged and that the internal signal was uneven. A necrotic cystic area with long T1 and T2 signals was seen in the center. The heterogeneous, solid T2 part of the tumor showed an uneven, long T2 signal. After injection of GD DTPA, the solid part of the mass was enhanced unevenly, and the necrotic cystic area was not obviously enhanced (Fig. 2A). Considering that the patient was 36 years old and the PSA value was 0.16 ng/mL, which was in the normal range (<4.0 ng/mL), and because of the lack of experience in diagnosing such cases, the local radiologist thought that this was benign prostatic disease. Two months later, the patient felt obvious dysuria, so he came to our hospital for treatment. On physical examination, a grade 3 enlarged prostate was found by digital rectal examination, the texture was hard, the surface was not smooth, the nodule could be touched, the central sulcus had become shallow, but there was no pressure pain, and a small amount of inflammatory secretion could be seen at the external orifice of the urethra. Urinalysis showed that urine RBCs had increased, with a value of 69.8/μL (0.00–25.00/μL), and a bacterial increase of 2111.3/μL (0.00–2000.0/μL). The serum PSA value was 0.18 ng/mL, which was in the normal range (<4.0 ng/mL). The patient was generally in good health and had no family genetic history or infectious history.

### 3.2 Imaging findings

Transrectal ultrasound showed that the prostate was 85 mm × 68 mm × 71 mm in size, and there was a fluid anechoic area with poor sound transmission. CDFI revealed a blood flow signal that could be seen in the solid part around the prostate. Elastic imaging showed that the texture of the solid part around the prostate was hard, and the texture of the central part was soft. Transrectal contrast-enhanced ultrasonography (TR-CEUS) showed that the necrotic area of the prostate was enlarged, and large perforating vessels could be seen in the necrotic area, which enhanced rapidly and synchronously with the prostate parenchyma (Fig. 2B). After routine disinfection, an 18 G PTC needle was used to penetrate into the center of the necrotic area, and 65 mL of dark red plasma was drawn out (Fig. 2C). Then, the solid part of the prostate, which was not necrotic according to contrast-enhanced ultrasound, was punctured and biopsied, and 10 tissue samples were obtained and sent to pathology, which confirmed Ewing's sarcoma of the prostate by immunohistochemistry. After that, the patient received PET/CT examination, and the results showed that the patient had multiple bone metastases in the 4th rib, 7th rib, and left iliac bone (Fig. 2D), as well as in the upper lobe of the right lung.

### 3.3 Operation and treatment

After the diagnosis was confirmed, because there was no contraindication to chemotherapy, the patient received cyclophosphamide and epirubicin; at the same time, acid suppression and nutritional support were carried out. After 6 cycles, the patient's condition was stable, and the right lung nodule had reduced in size, but the serum specific tissue polypeptide antigen (TPS) value remained high, with each test value greater than 140.94 U/L (normal range 0.00–80.00 U/L). However, the patient developed brain metastases 8 months later and died of brain hernia 1 year later.

## 4 Discussion

PS originates from prostatic stromal tissue and has high malignancy, early metastasis, rapid development, and a long course. PS is asymptomatic in the early stage, and most of its symptoms occur to the late stage. The clinical manifestations of PS depend on the degree of compression of the tumor on the surrounding rectum and urethra and mostly consist of progressive dysuria or dysuria.^[10]^ Tumor compression or invasion of the bladder or urethra can cause dysuria. Tumor compression of adjacent veins and nerves can cause lower extremity edema or pain. Hematuria is rare and often occurs when the mass is severely necrotic and liquefied. There are few abnormal findings in routine laboratory tests. Several scholars have reported that the common symptom of PSS is urinary retention, and the level of PSA in the blood often remains normal.^[11–14]^ On physical examination, the prostate gland is found to be swollen, unsmooth, nodular, and painless by digital rectal examination.

Imaging plays an important role in the treatment of patients with PS. It can detect not only enlargement of the prostate but also whether there are any bone, lung, and lymph node metastases. Imaging is very important for the diagnosis and staging of PS. A lack of understanding of imaging performance may hinder the diagnosis and treatment of these patients. Adrian et al reviewed and analyzed the MR images of 13 patients with PS and found that on T1 weighted images, the tumor often showed a low signal, while on the T2 weighted images, the tumor showed an uneven mass with medium and high signals. Necrosis and cystic changes are common in tumors and are related to rapid growth and high malignancy.^[15]^ Ultrasound examination is safe, convenient, and fast and can clearly show changes in the structure of the prostate parenchyma. In the above 2 cases, we found that the prostate was obviously enlarged. Because of the rapid growth of PS, necrosis and liquefaction usually appear in the interior of the prostate. Transrectal ultrasound can easily find this change. In the first case, transabdominal contrast-enhanced ultrasound was used, and in the second case, transrectal contrast-enhanced ultrasound was selected. Through contrast-enhanced ultrasound, we found that the PS had characteristic manifestations, including rapid enhancement of the prostate parenchyma on CEUS due to an abundant blood supply; and an area of cystic necrosis that appeared larger and with better quality on CEUS than on two-dimensional ultrasound. These findings are similar to those reported by Rena, who believed that adult PS was characteristically shown to be a large and heterogeneous mass with rapid, hypervascular, and heterogeneous enhancement on CT and MRI.^[16]^ With contrast-enhanced ultrasound, the solid part of the tumor could be clearly displayed, the necrosis and liquefaction area could be avoided, prostate puncture could be effectively guided, complete pathological tissues could be obtained, and a clear pathological diagnosis could be obtained. Therefore, CEUS could be used to evaluate adjuvant chemotherapy for patients with a late diagnosis who could not be treated by surgery.

In CEUS examination, PS needs to be differentiated from prostatic hyperplasia, prostatic abscess, and prostate cancer. PS occurs in the stroma of the prostate, and the detection of PSA and prostate-specific acid phosphatase in the normal range is helpful to distinguish PS from prostate cancer. In addition, regardless of whether two-dimensional ultrasound or CEUS is used, there are few large areas of necrosis and liquefaction in the prostate cancer focus. Furthermore, some nodular prostate cancer is significantly enhanced in the surrounding glands on contrast-enhanced ultrasound, which is helpful to distinguish between the 2. The rapid growth of PS needs to be differentiated from prostatic abscess. Prostatic abscess also has symptoms similar to PS, such as frequent urination, urgency of urination, dysuria, pain in defecation, etc. But the unique symptoms of prostatic abscess patients are obvious, such as fever, chills, etc. Tenderness of the prostate is obvious on digital examination of the rectum, and there are many purulent cells in microscopic examination of the prostatic fluid. In two-dimensional ultrasound examination, there are irregular transonic or hypoechoic areas in the prostate, and pus can be obtained by puncture.

### 4.1 Lessons

PS showed diffuse enlargement of the prostate on sonography, and the necrotic liquefying area within the large vessels could be clearly displayed by CEUS. Considering the characteristic features of PS in CEUS, CEUS can be advocated as a valuable noninvasive and safe imaging diagnosis method.

## Author contributions

**Funding acquisition:** Hua Hong, Guozhu Wu.

**Resources:** Ran Sun, Aitong Sun.

**Software:** Jing Li, Qian Liu.

**Ultrasonic examination:** Yumin Wang,Guozhu Wu, Hua Hong, Ran Sun.

**Writing – original draft:** Guozhu Wu.

**Writing – review & editing:** Hua Hong, Guozhu Wu, Yumin Wang.

All authors reviewed and approved the final draft of the manuscript.

## Abbreviations

Abbreviations: CDFI = color Doppler flow imaging, CEUS = contrast-enhanced ultrasonography, CT = computed tomography, MRI = magnetic resonance imaging, PS = prostatic sarcoma, PSA = prostate-specific antigen.
